# Supplementary material for: Enterococcus faecalis biofilm rewires neutrophil metabolism to suppress antimicrobial activity
Source: Sci Adv. 2026 Oct 21;12(43):eaej4153. doi: 10.1126/sciadv.aej4153 (PMC13685926; doi:10.1126/sciadv.aej4153)
Supplement: Supplementary file 1 [file sciadv.aej4153_sm.pdf]

Science Advances
AAAS

Supplementary Materials for

Enterococcus faecalis biofilm rewires neutrophil metabolism to suppress antimicrobial activity

Haris Antypas *et al.*

Corresponding author: Kimberly A. Kline, kimberly.kline@unige.ch

Sci. Adv. **12**, eaej4153 (2026)
DOI: 10.1126/sciadv.aej4153

The PDF file includes:

Figs. S1 to S10
Table S1
Legends for data files S1 to S3
References

Other Supplementary Material for this manuscript includes the following:

Data files S1 to S3

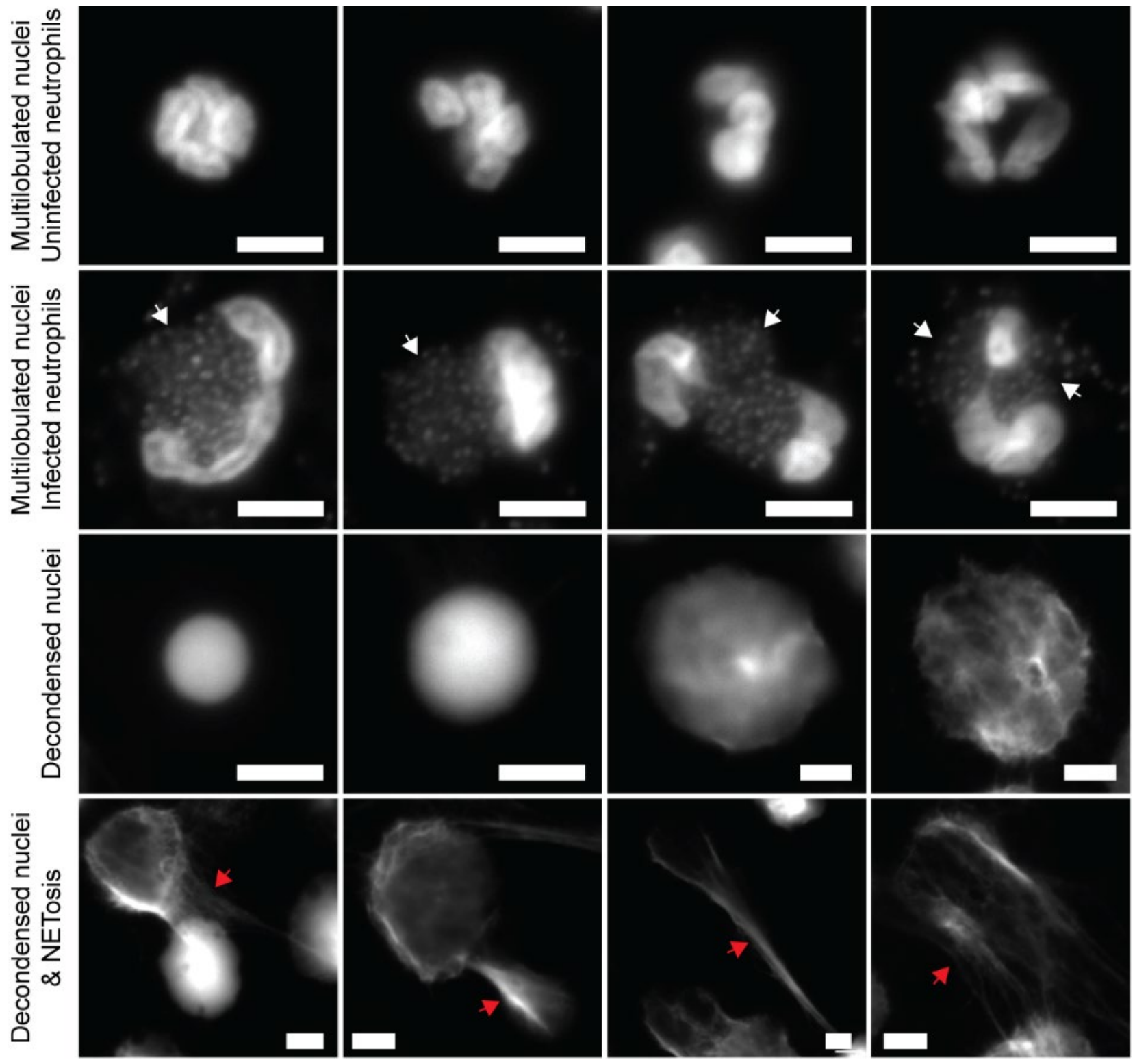

Supplementary Figure S1. Representative neutrophil nuclear morphologies used for NETosis classification. DAPI-stained fluorescence microscopy images showing the morphological criteria used to classify neutrophil nuclei. Images were captured using a Zeiss Axio Observer Z1 inverted widefield microscope with a 63× oil immersion lens. Uninfected neutrophils with compact or loose multilobulated nuclei were classified as non-decondensed nuclei. In infected neutrophils, multilobulated nuclei may appear morphologically altered because intracellular bacteria (white arrows) occupy cellular volume and can displace or separate nuclear lobes across different focal planes. Although these nuclei may deviate from the appearance of uninfected neutrophil nuclei, they were still classified as non-decondensed nuclei when nucleus lobulation was retained. Neutrophils showing loss of multilobulated nuclear structure, rounding, and nuclear swelling were classified as having decondensed nuclei. Cells with decondensed nuclei associated with extracellular DNA fibers were classified as nuclei releasing NETs. Red arrows = NETs, white arrows = bacteria, scale bar = 5 µm.

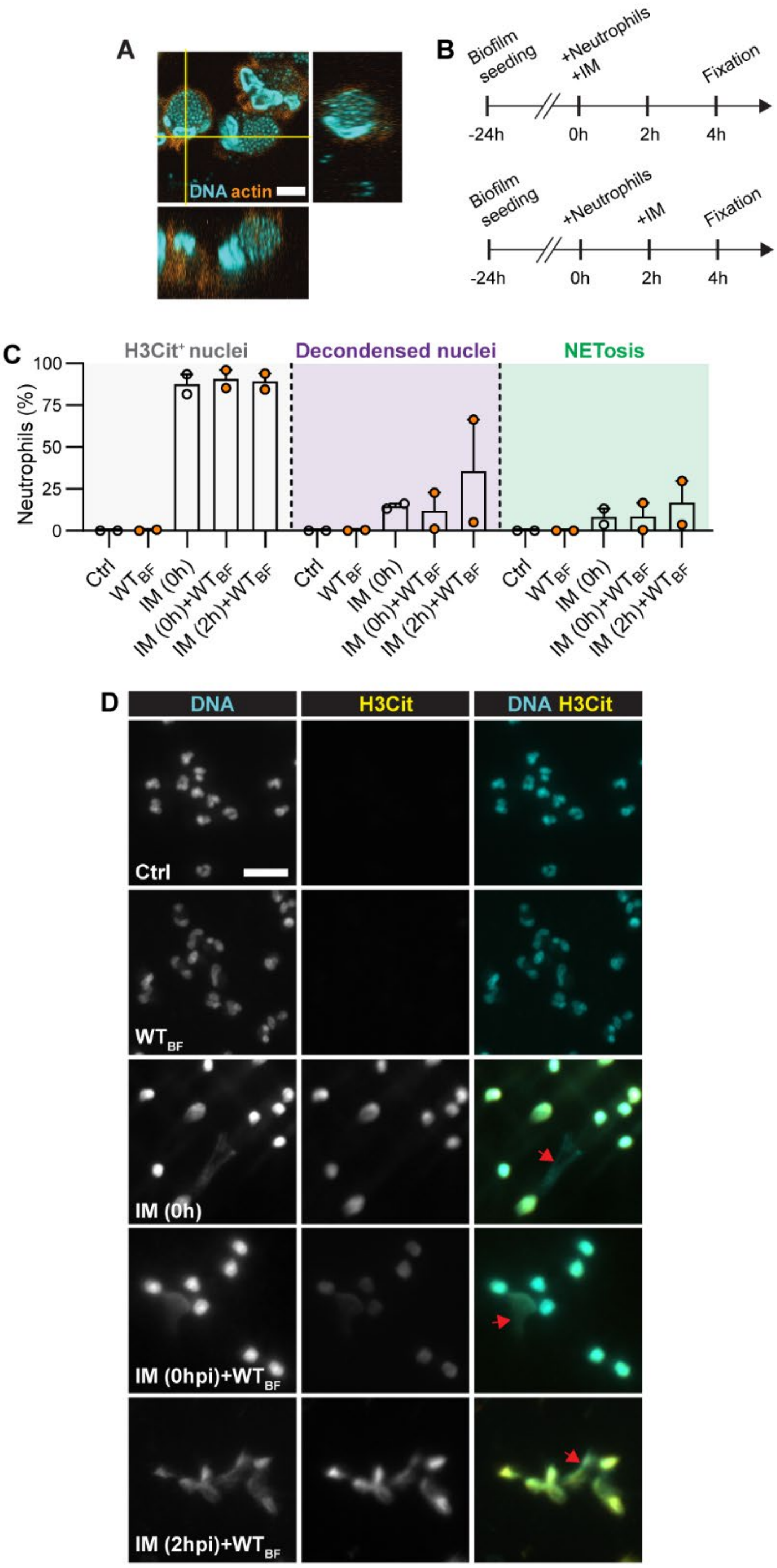

Supplementary Figure S2. Ionomycin-mediated NETosis in human neutrophils is not completely inhibited by *E. faecalis* biofilm. A. Human neutrophils with internalized *E. faecalis* after exposure to biofilm for 4 h. Representative single section with orthogonal views from a Z-stack, stained for DNA and F-actin. N = 3, Scale = 5 µm. **B.** Experimental timeline for C-D. Biofilm was grown for 24 h, followed by either simultaneous addition of human neutrophils and ionomycin (IM; 4 µM) at 0 h or addition of neutrophils at 0 h and IM at 2 h, followed by fixation at 4 h. **C-D.** Quantification (C) and representative immunofluorescence microscopy images (D) of NETosis hallmarks in neutrophils stained with DAPI and anti-H3Cit antibody. The percentage of H3Cit^{+} nuclei, decondensed nuclei, and NET-releasing cells (NETosis) was

quantified from N = 2 independent experiments and is shown as mean ± SEM. Scale bar = 20 µm; red arrow = NETs. Ctrl = uninfected & unstimulated neutrophils in media. The control sample from one donor shown in panel C is the same control samples in Fig. S10A, as both panels were generated from neutrophils deriving from the same blood donation.

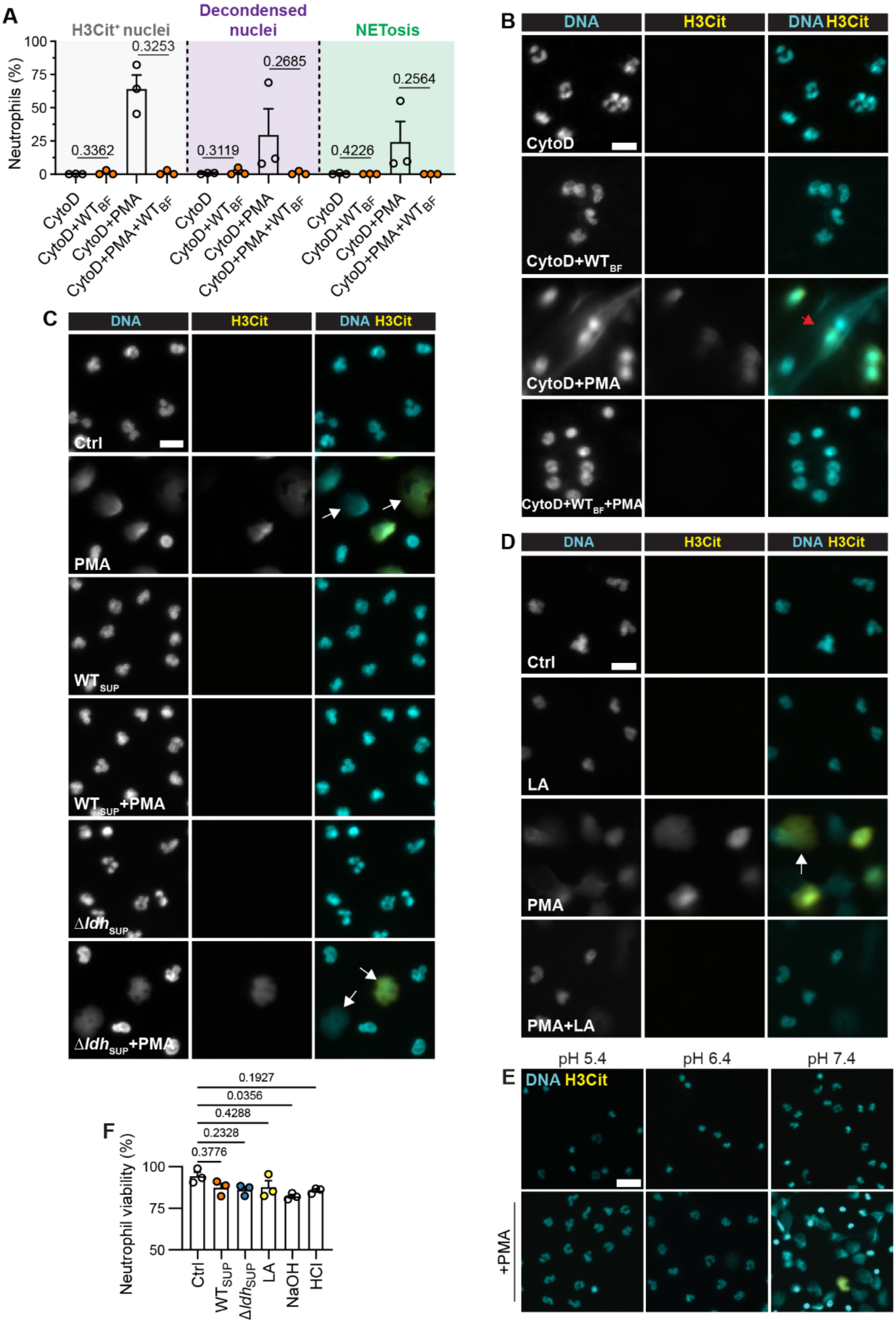

Supplementary Figure S3. ***E. faecalis*** **inhibits NETosis via lactic acid-mediated extracellular acidification. A.** Quantification of NETosis hallmarks from immunofluorescence microscopy images. Mean (%) citrullinated histone H3 stained nuclei (H3Cit^{+}), decondensed nuclei, and NETs release (NETosis) from N = 3 independent experiments are shown, error = SEM. Human neutrophils were pre-incubated with Cytochalasin D (30 µM; CytoD) for 30 min prior to

addition to the biofilm, stimulated with PMA at 0 h and incubated with *E. faecalis* OG1RF biofilm for 4 h in the continued presence of CytoD. CytoD and cytoD + PMA control samples shown for one donor in panel A are the same control samples shown in Fig. 5A, as one biological replicate for both panels was generated from neutrophils from the same blood donation. Statistical significance was assessed with two-tailed paired t-test. **B-E**. Representative immunofluorescence microscopy images of neutrophils stained with DAPI and anti-H3Cit antibody. N = 3. Fig. S3B corresponds to quantification shown in Fig. S3A, Fig. S3C to Fig. 2A, Fig. S3D to Fig. 2B, Fig. S3E to Fig. 2D. Scale = 10 µm in Fig. S3B-D and 20 µm in Fig. S3E, red arrow = NETs, white arrow = decondensed nuclei. Only merged images shown in Fig. S3E. **F.** Quantification of neutrophil viability by propidium iodide 4 h after exposure to the indicated conditions. Fluorescence values were normalized to a positive control of neutrophils treated with 0.1% Triton X-100. N = 3, error = SEM. Statistical significance was assessed with one-way ANOVA followed by Tukey's multiple comparisons test; $\alpha = 0.05$ with significant values shown in blue.

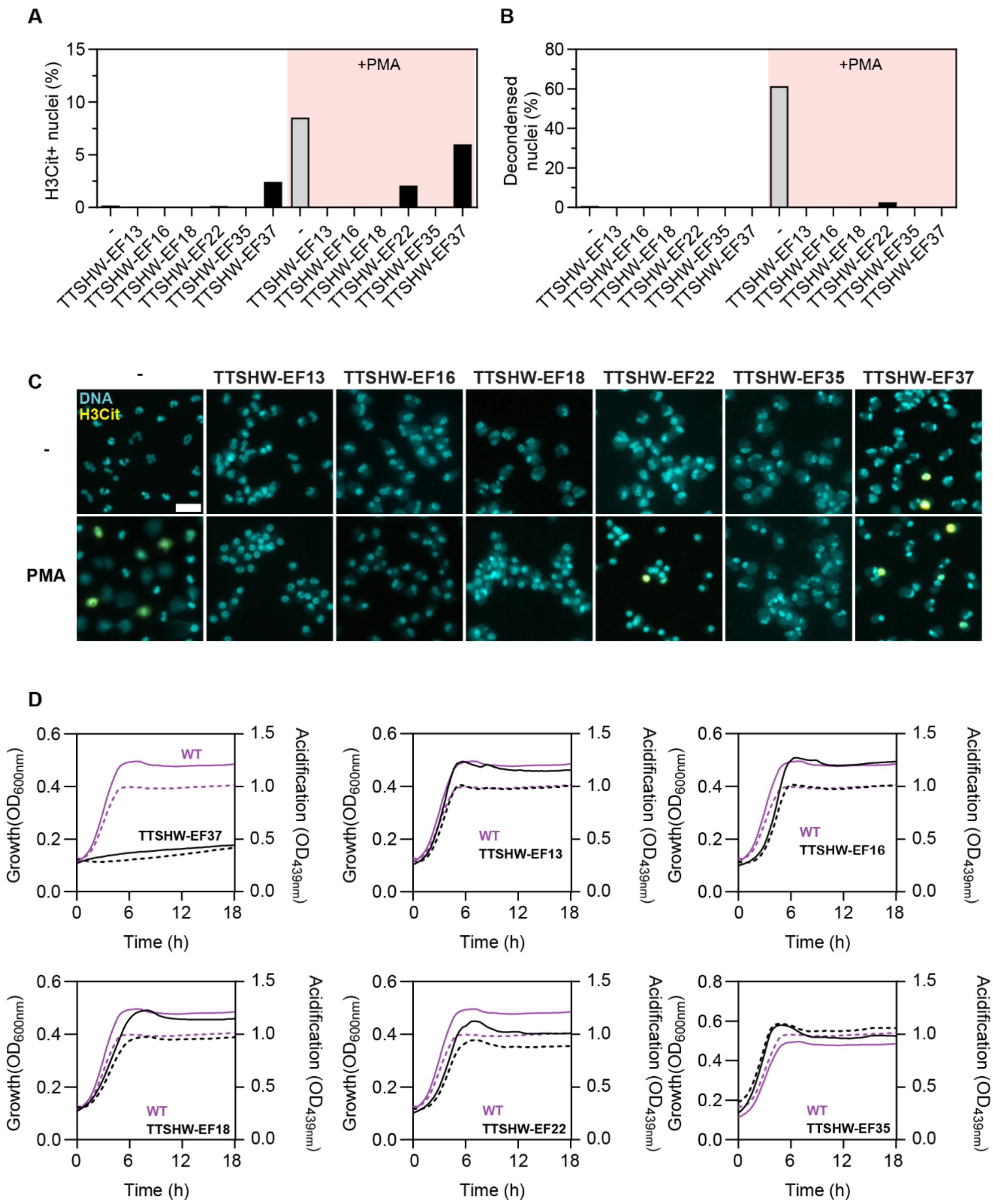

Supplementary Figure S4. Effect of *E. faecalis* clinical isolates on NETosis. A-C. Human neutrophils were incubated with *E. faecalis* clinical isolates biofilm for 4 h and stimulated with PMA (100 nM) at 0 h. NETosis was assessed by immunofluorescence microscopy based on nuclear decondensation and citrullinated histone H3 (H3Cit) staining. Mean (%) of H3Cit positive and decondensed nuclei is shown together with representative images from N = 1 experiment. Scale = 20 µm. **D.** Growth curves (solid line) based on OD_{600nm} measurements (left Y-axis) and acidification curves (dashed line) based on OD_{439nm} measurements (right Y-axis) of *E. faecalis* clinical isolates in IMDM**.** N = 1.

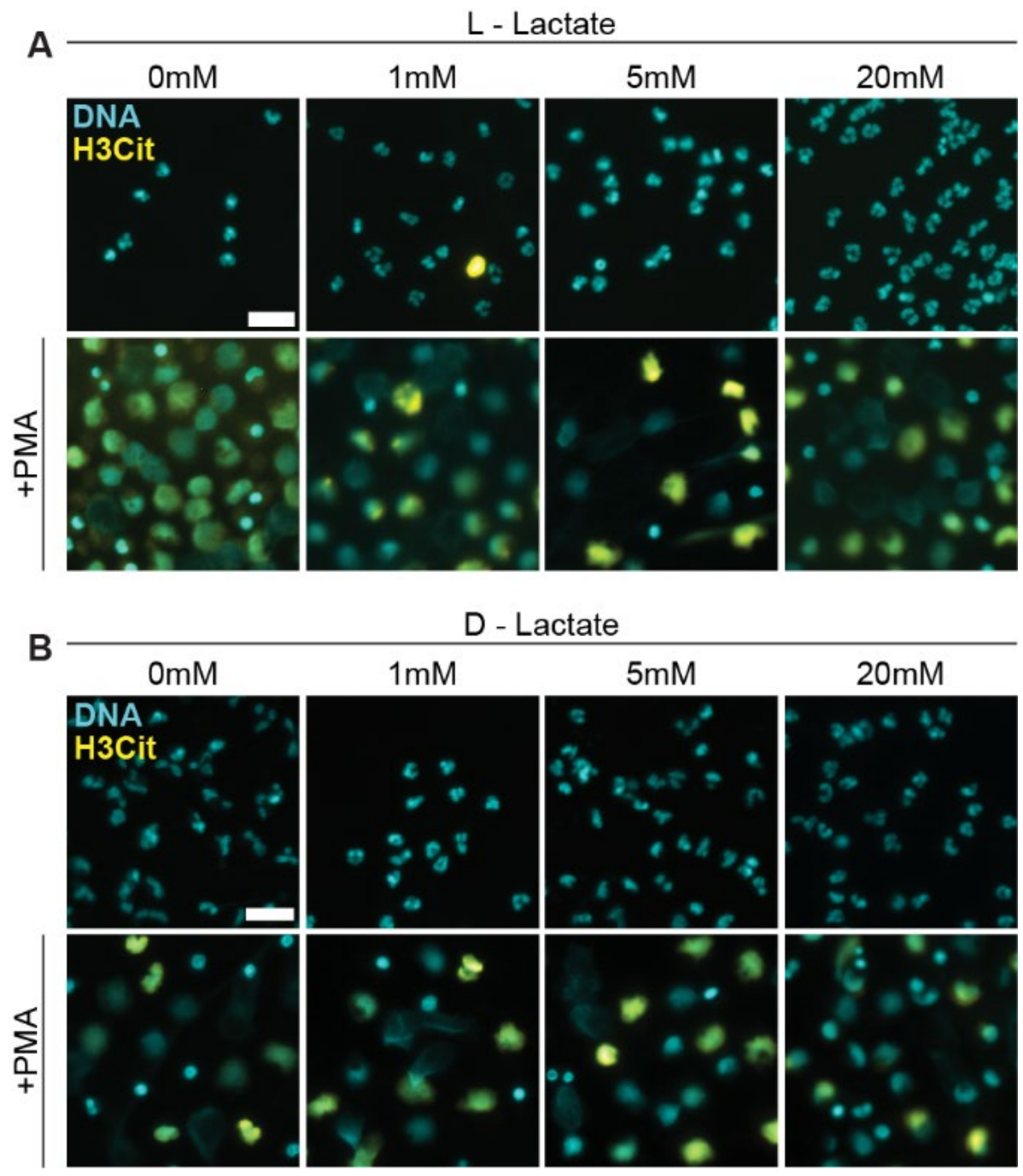

Supplementary Figure S5. L- and D-lactate do not induce NETosis in human neutrophils. A-B. Representative immunofluorescence microscopy images of neutrophils stained with DAPI and anti-H3Cit antibody from N = 3, with corresponding NETosis quantification shown in **Fig. 2E-F.** Human neutrophils were incubated for 4 h and stimulated with sodium L- or D-lactate and PMA at 0 h. Scale = 20 µm. Only merged images shown.

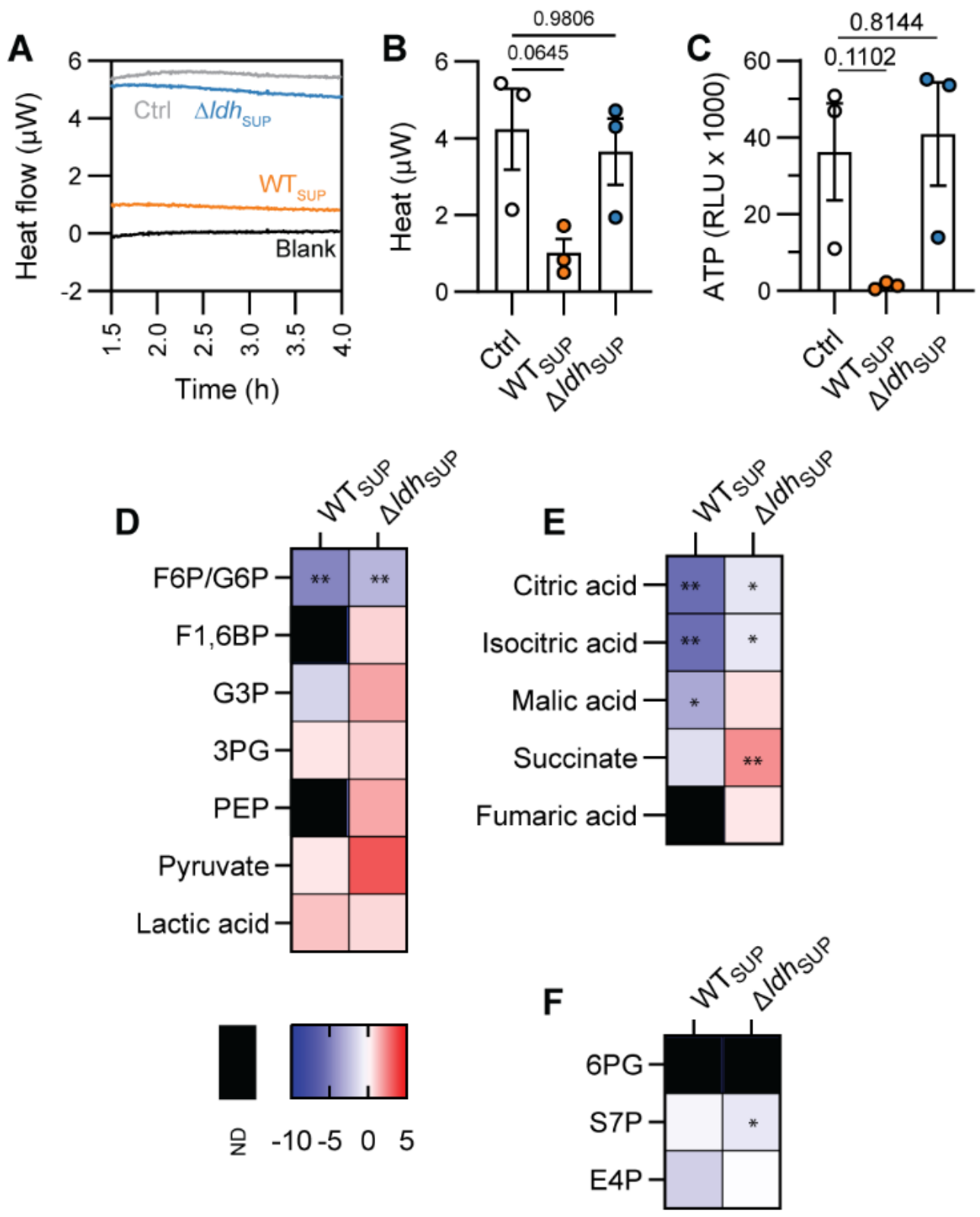

Supplementary Figure S6. *E. faecalis*-derived lactic acid suppresses glycolysis, TCA cycle, and oxPPP in dHL-60 cells. A. Representative thermogram of dHL-60 cells treated with fresh media (Ctrl), or biofilm supernatant from WT (WT_{SUP}) or Δ*ldh* (Δ*ldh*$_{SUP}$) for 4 h. Media alone (blank) was included. Heat measurements start after 1.5 h due to the calScreener initial calibration phase. N = 3. **B.** Mean heat produced by dHL-60 cells at 4 h post-incubation. N = 3, error = SEM. Statistical significance was assessed with paired t-test. **C.** ATP levels in dHL-60 cells after incubation with media alone (Ctrl), WT_{SUP} or Δ*ldh*$_{SUP}$ for 4 h. Data is presented as relative luminescence units, N = 3, error = SEM. Statistical significance was assessed with two-tailed Welch's test. **D-F.** LC-MS heatmaps showing log2 fold changes in metabolite abundance in dHL-60 cells after exposure to WT_{SUP} or Δ*ldh*$_{SUP}$ for 4 h relative to untreated control cells. Metabolites from glycolysis (D), TCA cycle (E), and PPP (F) are shown. N = 3, color scale indicates relative metabolite abundance, with red denoting increased and blue decreased levels relative to control, and black denoting not detected (ND) metabolites. Statistics were calculated on the biological replicate mean peak areas using a two-tailed Welch's t-test to compare WT_{SUP} with media and Δ*ldh*$_{SUP}$ with media for each metabolite. * = P < 0.05, ** = P < 0.01, *** = P < 0.001, **** = P < 0.0001; α = 0.05 with significant values shown in blue.

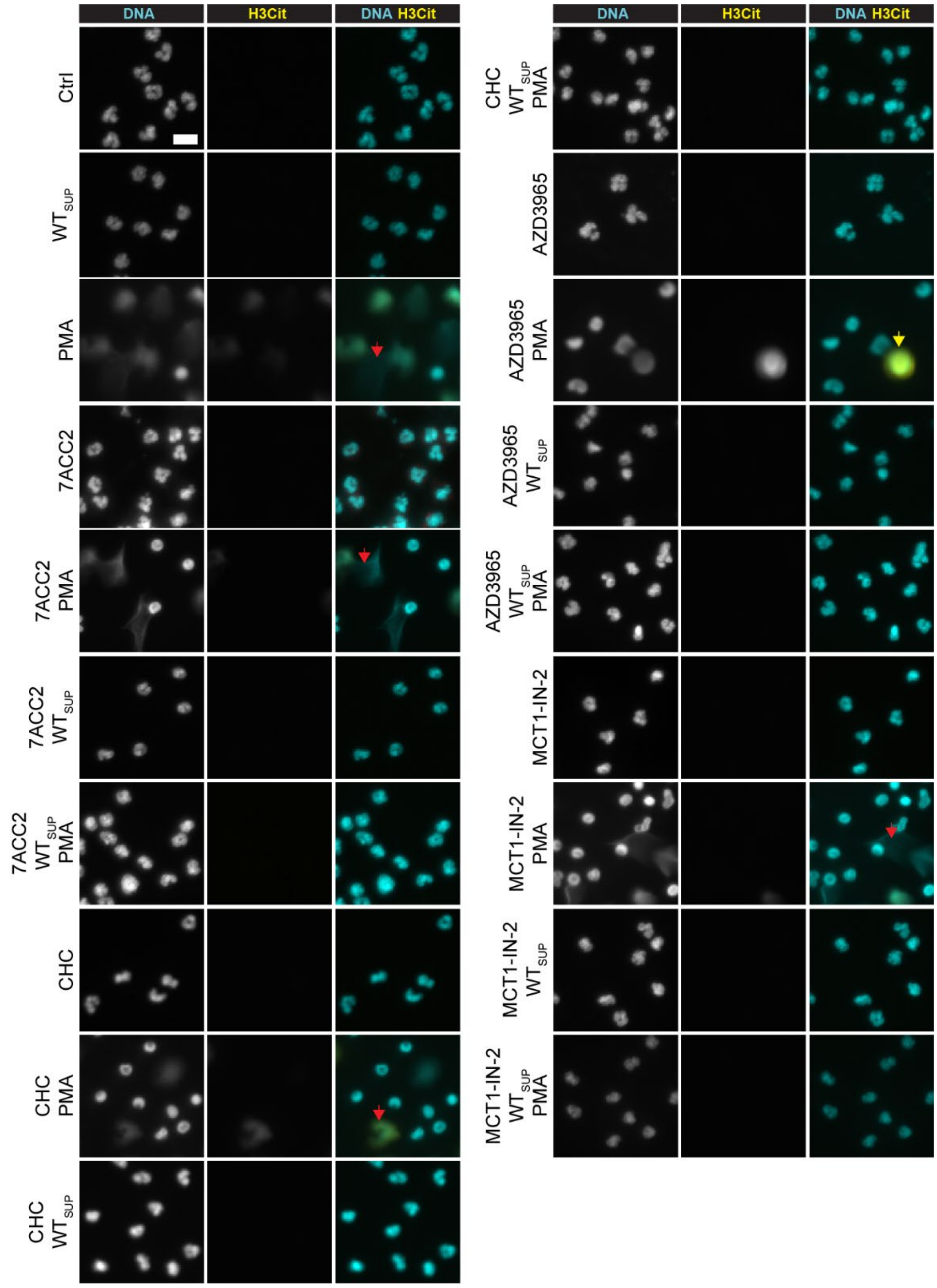

Supplementary Figure S7. MCT inhibitors partially inhibit PMA-induced NETosis. Human neutrophils were stimulated with PMA (100 nM) at 0 h and incubated with *E. faecalis* OG1RF WT biofilm supernatant (WT_{SUP}) and MCT inhibitors 7ACC2 (20 µM), CHC (40 µM), AZD3965 (25 nM), MCT1-IN-2 (100 nM) for 4 h. NETosis was assessed by immunofluorescence microscopy based on nuclear decondensation and citrullinated histone H3 (H3Cit) staining. Red arrows = NETs, yellow arrow = decondensed nuclei. Representative images are shown from N = 1. Scale = 10 µm.

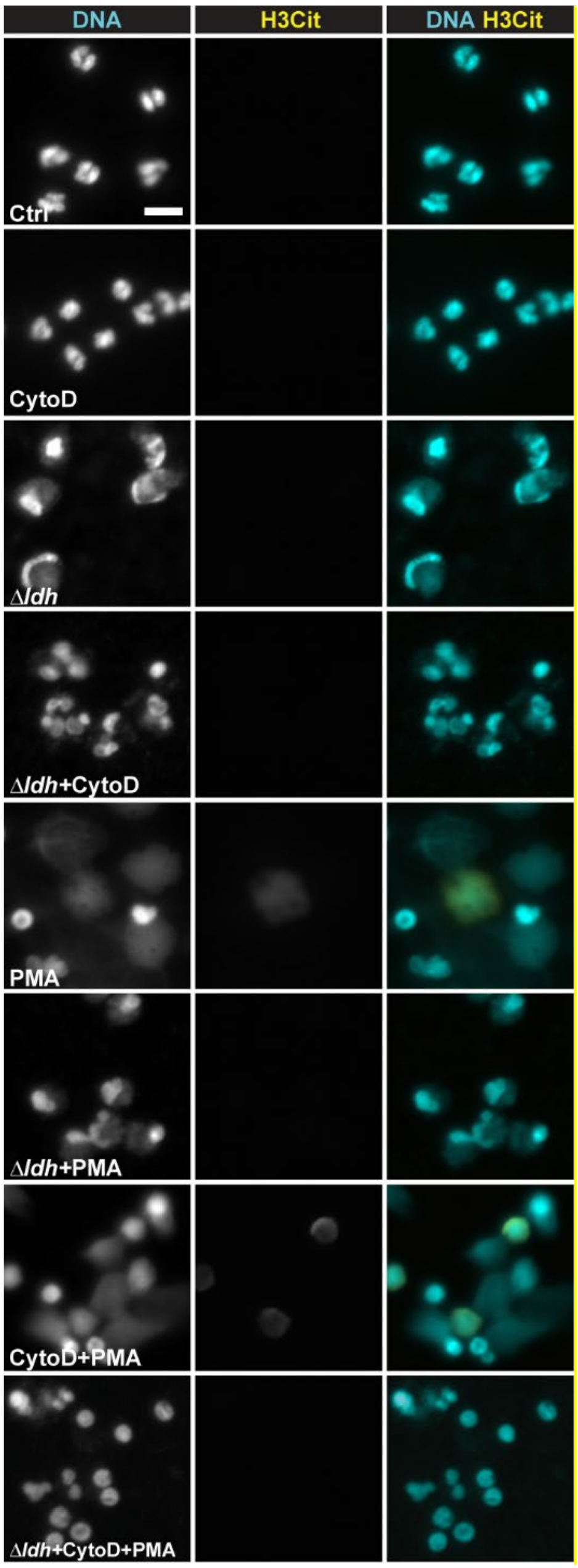

Supplementary Figure S8. Δ*ldh* biofilm inhibits PMA-induced NETosis in neutrophils extracellularly. Representative fluorescence microscopy images of human neutrophils incubated with Δ*ldh* biofilm and supplemented with cytochalasin D (cytoD; 30 μM) and PMA (100 nM) as indicated at 4 h post-incubation, corresponding to quantified data in Fig. 5A. Neutrophil nuclei were stained with DAPI and an antibody against citrullinated histone H3 (H3Cit). N = 3, scale = 10 μm.

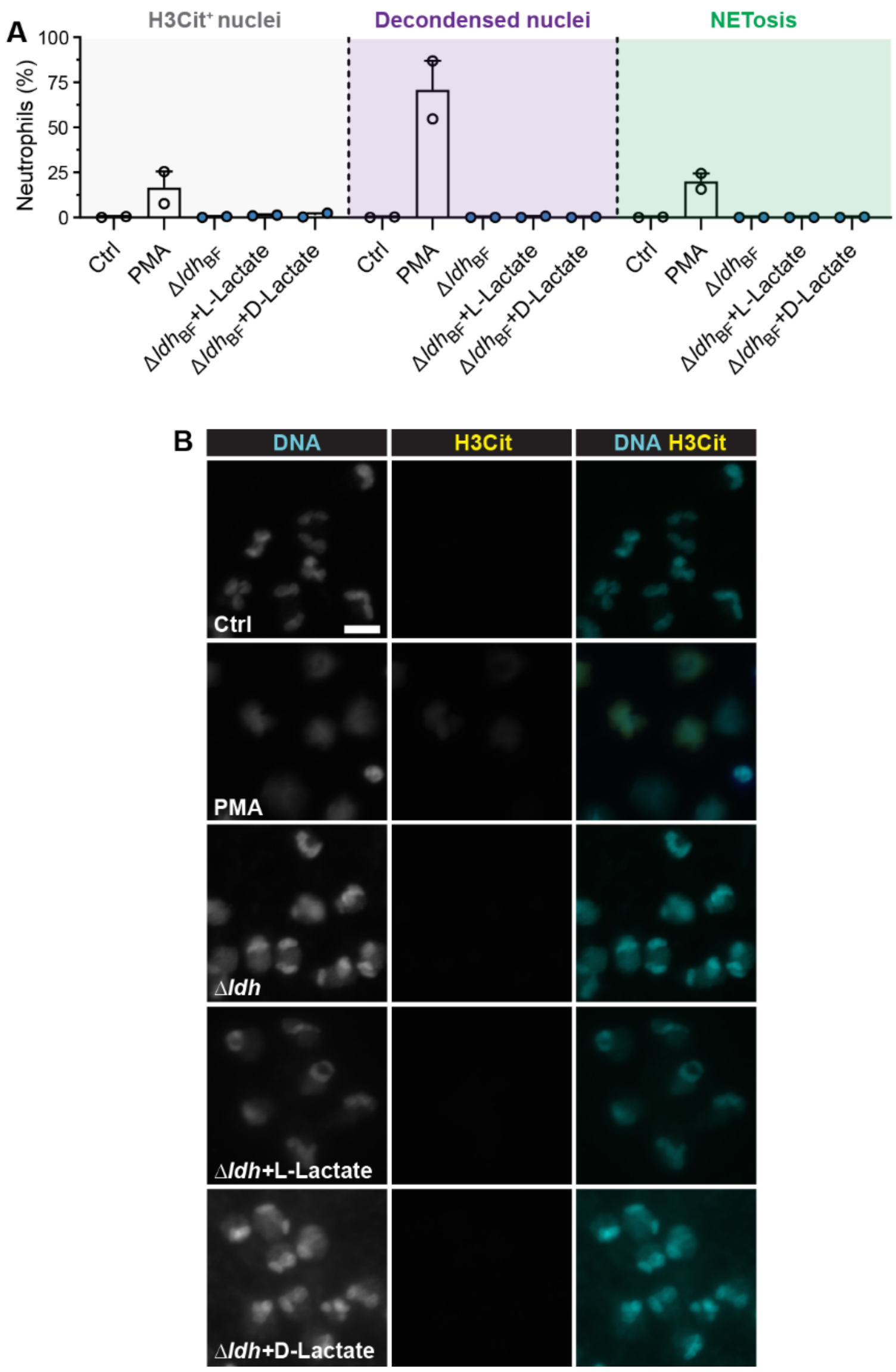

Supplementary Fig. S9. Addition of L- or D-lactate to Δ*ldh* biofilms does not induce NETosis in human neutrophils. A-B. Quantification of NETosis hallmarks from immunofluorescence microscopy images. Mean (%) citrullinated histone H3 stained nuclei (H3Cit$^+$), decondensed nuclei, and NETs release (NETosis) from N = 2 independent experiments are shown, alongside representative images. Human neutrophils were supplemented with 20mM of L- or D-lactate at 0 h and incubated with *E. faecalis* OG1RF Δ*ldh* biofilm for 4 h. Ctrl = neutrophils in media, error = SEM, scale = 10 μm.

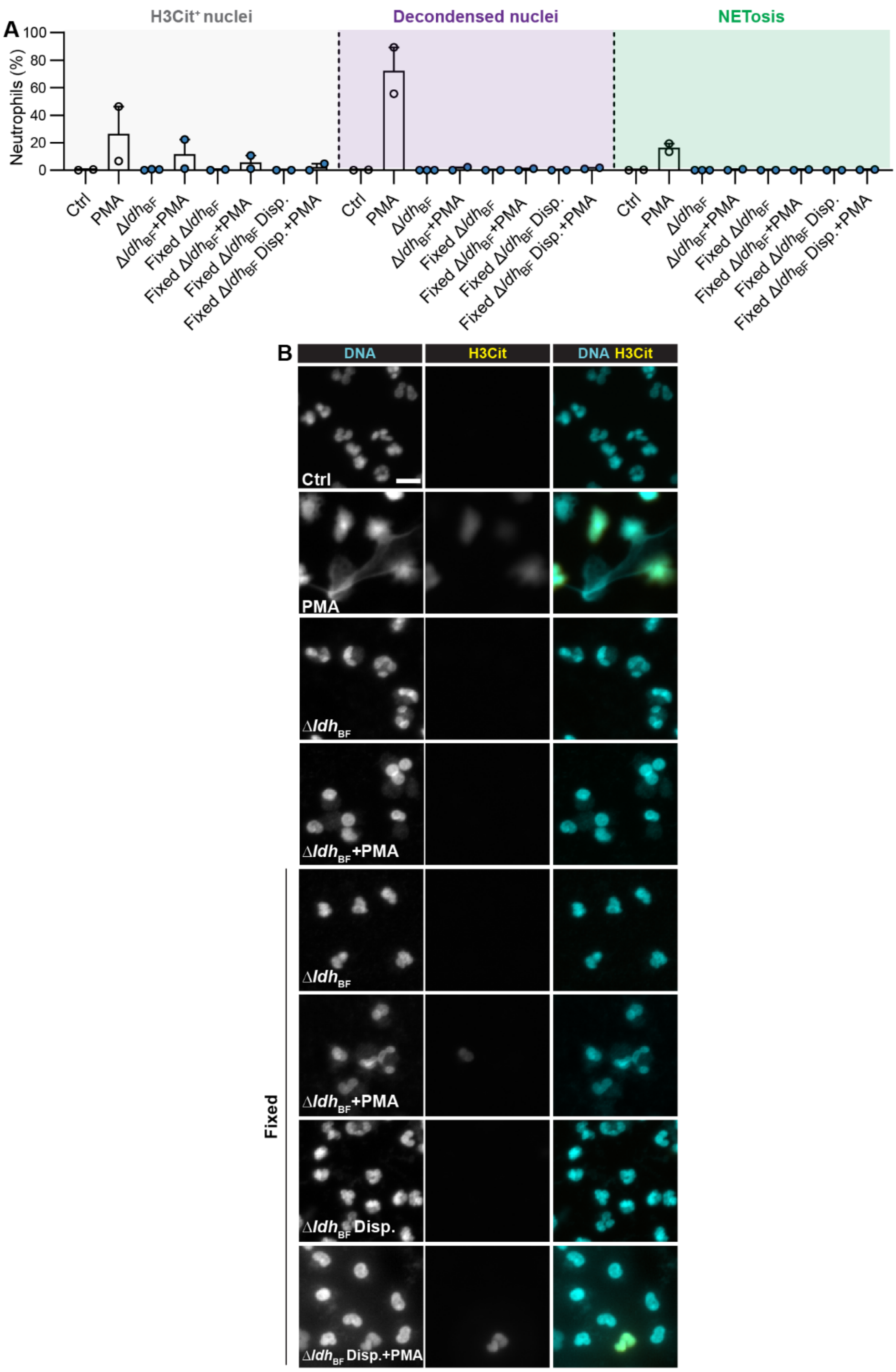

Supplementary Figure S10. PFA-fixed Δ*ldh* biofilm suppresses NETosis. A. Quantification of NETosis hallmarks from immunofluorescence microscopy images. Mean (%) citrullinated histone H3 stained nuclei ($H3Cit^+$), decondensed nuclei, and NETs release (NETosis) from N = 2 independent experiments are shown, error = SEM. Human neutrophils were incubated with live, PFA-fixed or dispersed (Disp.) Δ*ldh* biofilm (Δ*ldh*$_{BF}$) in the presence of PMA (100 nM) as indicated for 4 h. The control sample shown for one donor in panel A is shared with Fig. S2C, as one replicate from both panels was generated from neutrophils deriving from the same

blood donation. **B.** Representative images of neutrophils for S10A with nuclei stained for DAPI and citrullinated histone H3 (H3Cit). N = 2-3, scale = 10 µm.

Table S1. Bacterial strains

Strain	Description	Reference
OG1RF	*Enterococcus faecalis, Rif^R, Fus^R*	(73)
Δ*ldh*	OG1RF *ldh1* and *ldh2* deletion	(15)
WT Pcfb-GFP	OG1RF WT with constitutively expressed Dasher GFP	(74)
TTSHW-EF13	*Enterococcus faecalis* isolate from buttock wound, *cps*$^+$, *fsr*$^+$	(75)
TTSHW-EF16	*Enterococcus faecalis* isolate from wound swab, *esp*$^+$	(75)
TTSHW-EF18	*Enterococcus faecalis* isolate from sacral swab, *cyl*$^+$, *cps*$^+$, *fsr*$^+$	(75)
TTSHW-EF22	*Enterococcus faecalis* isolate from wound swab, *cyl*$^+$, *cps*$^+$, *esp*$^+$	(10)
TTSHW-EF35	*Enterococcus faecalis* isolate from leg wound, *fsr*$^+$	This study
TTSHW-EF37	*Enterococcus faecalis* isolate from peripancreatic fluid, *cyl*+, *esp*+	This study

Supplementary Data File captions

Data File S1. LC-MS analysis of lysates from human neutrophils exposed to media, WT and Δldh biofilm supernatant for 4h.

Data File S2. LC-MS analysis of lysates from dHL-60 cells exposed to media, WT and Δldh biofilm supernatant for 4h.

Data File S3. Source data file containing the underlying data for Figures 1 to 6 and Supplementary Figures S2, S3, S4, S6, S9, and S10.

REFERENCES

1. E. Fiore, D. Van Tyne, M. S. Gilmore, Pathogenicity of enterococci. *Microbiol. Spectr.* **7**, 10.1128/microbiolspec.gpp3-0053-2018 (2019).

2. J.-H. Ch'ng, K. K. L. Chong, L. N. Lam, J. J. Wong, K. A. Kline, Biofilm-associated infection by enterococci. *Nat. Rev. Microbiol.* **17**, 82–94 (2019).

3. P. H. N. Kao, K. A. Kline, Dr. Jekyll and Mr. Hide: How *Enterococcus faecalis* subverts the host immune response to cause infection. *J. Mol. Biol.* **431**, 2932–2945 (2019).

4. K. K. L. Chong, W. H. Tay, B. Janela, A. M. H. Yong, T. H. Liew, L. Madden, D. Keogh, T. M. S. Barkham, F. Ginhoux, D. L. Becker, K. A. Kline, *Enterococcus faecalis* modulates immune activation and slows healing during wound infection. *J. Infect. Dis.* **216**, 1644–1654 (2017).

5. H. Antypas, V. Schmidtchen, W. I. Staiger, L. I. Yanhong, R. J. W. Tan, K. K. F. Ng, C. J. Y. Neo, S. M. Radhesh, F. R. Tanoto, R. A. G. da Silva, C. Colomer-Winter, S. D. Schütz, J. Kloehn, L. Muthualagu Natarajan, C. Manzano, J. J. Wong, K. Pethe, B. Hasse, S. D. Brugger, S. L. Wong, D. Van Tyne, A. S. Zinkernagel, K. A. Kline, Loss of Fsr quorum sensing promotes biofilm formation and worsens outcomes in enterococcal infective endocarditis. *Nat. Commun.* **17**, 1668 (2026).

6. P. S. Guiton, T. J. Hannan, B. Ford, M. G. Caparon, S. J. Hultgren, *Enterococcus faecalis* overcomes foreign body-mediated inflammation to establish urinary tract infections. *Infect. Immun.* **81**, 329–339 (2013).

7. M. Rousseau, H. M. S. Goh, S. Holec, M. L. Albert, R. B. Williams, M. A. Ingersoll, K. A. Kline, Bladder catheterization increases susceptibility to infection that can be prevented by prophylactic antibiotic treatment. *JCI Insight* **1**, e88178 (2016).

8. C. Archambaud, N. Nunez, R. A. G. da Silva, K. A. Kline, P. Serror, *Enterococcus faecalis*: An overlooked cell invader. *Microbiol. Mol. Biol. Rev.* **88**, e0006924 (2024).

9. R. A. G. Da Silva, W. H. Tay, F. K. Ho, F. R. Tanoto, K. K. L. Chong, P. Y. Choo, A. Ludwig, K. A. Kline, *Enterococcus faecalis* alters endo-lysosomal trafficking to replicate and persist within mammalian cells. *PLOS Pathog.* **18**, e1010434 (2022).

10. C. J. Stocks, R. A. G. da Silva, H. Antypas, N. Jeyabalan, S. L. Wong, K. A. Kline, *Enterococcus faecalis* persists and replicates intracellularly within neutrophils. *Infect. Immun.* **94**, e0036425 (2026).

11. F. R. Tanoto, J. H. Liew, C. J. Stocks, D. Rawat, K. K. L. Chong, K. Pethe, H. Antypas, K. A. Kline, Gelatinase regulates the egress of intracellular replicating populations during *Enterococcus faecalis* infection. *PLOS Pathog.* **22**, e1013738 (2026).

12. R. M. Rakita, N. N. Vanek, K. Jacques-Palaz, M. Mee, M. M. Mariscalco, G. M. Dunny, M. Snuggs, W. B. Van Winkle, S. I. Simon, *Enterococcus faecalis* bearing aggregation substance is resistant to killing by human neutrophils despite phagocytosis and neutrophil activation. *Infect. Immun.* **67**, 6067–6075 (1999).

13. S. D. Sussmuth, A. Muscholl-Silberhorn, R. Wirth, M. Susa, R. Marre, E. Rozdzinski, Aggregation substance promotes adherence, phagocytosis, and intracellular survival of *Enterococcus faecalis* within human macrophages and suppresses respiratory burst. *Infect. Immun.* **68**, 4900–4906 (2000).

14. B. Y. Q. Tien, H. M. S. Goh, K. K. L. Chong, S. Bhaduri-Tagore, S. Holec, R. Dress, F. Ginhoux, M. A. Ingersoll, R. B. H. Williams, K. A. Kline, *Enterococcus faecalis* promotes innate immune suppression and polymicrobial catheter-associated urinary tract infection. *Infect. Immun.* **85**, e00378-17 (2017).

15. R. A. G. da Silva, B. Y. Q. Tien, P. H. N. Kao, H. Antypas, C. Celik, A. Z. C. Tan, M. H. Ismail, G. Hu, K. K. L. Chong, G. Thibault, J. Chen, K. A. Kline, *Enterococcus faecalis*-derived lactic acid suppresses macrophage activation to facilitate persistent and polymicrobial wound infections. *Cell Host Microbe* **34**, 245–262.e8 (2026).

16. H. R. Thiam, S. L. Wong, D. D. Wagner, C. M. Waterman, Cellular mechanisms of NETosis. *Annu. Rev. Cell Dev. Biol.* **36**, 191–218 (2020).

17. P. H.-N. Kao, J.-H. Ch'ng, K. K. L. Chong, C. J. Stocks, S. L. Wong, K. A. Kline, *Enterococcus faecalis* suppresses *Staphylococcus aureus*-induced NETosis and promotes bacterial survival in polymicrobial infections. *FEMS Microbes* **4**, xtad019 (2023).

18. E. Tamrat, Z. Asmare, A. Geteneh, A. Sisay, E. Getachew, B. Kassanew, M. Dessale, Y. Gashaw, A. Jemal, M. Gashaw, A. Bazezew, S. Gedfie, W. Kassahun, W. Abebe, Z. Dejazmach, T. Misganaw, A. Ashagre, M. Nigatie, A. A. Damtie, B. B. Alemu, Z. Tefera, B. Mezgebu, G. Kumie, M. Kiros, M. A. Reta, The global prevalence of biofilm-forming *Enterococcus faecalis* in clinical isolates: A systematic review and meta-analysis. *BMC Infect. Dis.* **25**, 981 (2025).

19. A. M. T. Barnes, J. L. Dale, Y. Chen, D. A. Manias, K. E. Greenwood Quaintance, M. K. Karau, P. C. Kashyap, R. Patel, C. L. Wells, G. M. Dunny, *Enterococcus faecalis* readily colonizes the entire gastrointestinal tract and forms biofilms in a germ-free mouse model. *Virulence* **8**, 282–296 (2017).

20. P. S. Guiton, C. S. Hung, L. E. Hancock, M. G. Caparon, S. J. Hultgren, Enterococcal biofilm formation and virulence in an optimized murine model of foreign body-associated urinary tract infections. *Infect. Immun.* **78**, 4166–4175 (2010).

21. A. M. T. Barnes, K. S. Ballering, R. S. Leibman, C. L. Wells, G. M. Dunny, *Enterococcus faecalis* produces abundant extracellular structures containing DNA in the absence of cell lysis during early biofilm formation. *mBio* **3**, e00193-12 (2012).

22. Y. Guerardel, I. Sadovskaya, E. Maes, S. Furlan, M. P. Chapot-Chartier, S. Mesnage, L. Rigottier-Gois, P. Serror, Complete structure of the enterococcal polysaccharide antigen (EPA) of vancomycin-resistant *Enterococcus faecalis* v583 reveals that EPA decorations are teichoic acids covalently linked to a rhamnopolysaccharide backbone. *mBio* **11**, e00277-20 (2020).

23. F. Teng, K. V. Singh, A. Bourgogne, J. Zeng, B. E. Murray, Further characterization of the epa gene cluster and epa polysaccharides of *Enterococcus faecalis*. *Infect. Immun.* **77**, 3759–3767 (2009).

24. Y. Ramos, J. Rocha, A. L. Hael, J. van Gestel, H. Vlamakis, C. Cywes-Bentley, J. R. Cubillos-Ruiz, G. B. Pier, M. S. Gilmore, R. Kolter, D. K. Morales, PolyGlcNAc-containing exopolymers enable surface penetration by non-motile *Enterococcus faecalis*. *PLOS Pathog.* **15**, e1007571 (2019).

25. A. Taglialegna, L. Matilla-Cuenca, P. Dorado-Morales, S. Navarro, S. Ventura, J. A. Garnett, I. Lasa, J. Valle, The biofilm-associated surface protein Esp of *Enterococcus faecalis* forms amyloid-like fibers. *NPJ Biofilms Microbiomes* **6**, 15 (2020).

26. P. M. Tendolkar, A. S. Baghdayan, M. S. Gilmore, N. Shankar, Enterococcal surface protein, Esp, enhances biofilm formation by *Enterococcus faecalis*. *Infect. Immun.* **72**, 6032–6039 (2004).

27. J. Hirschfeld, Dynamic interactions of neutrophils and biofilms. *J. Oral Microbiol.* **6**, 26102 (2014).

28. M. Bhattacharya, E. T. M. Berends, R. Chan, E. Schwab, S. Roy, C. K. Sen, V. J. Torres, D. J. Wozniak, *Staphylococcus aureus* biofilms release leukocidins to elicit extracellular trap formation and evade neutrophil-mediated killing. *Proc. Natl. Acad. Sci. U.S.A.* **115**, 7416–7421 (2018).

29. C. A. Z. Tan, L. N. Lam, G. Biukovic, E. Y. C. Soh, X. W. Toh, J. A. Lemos, K. A. Kline, *Enterococcus faecalis* antagonizes *Pseudomonas aeruginosa* growth in mixed-species interactions. *J. Bacteriol.* **204**, e0061521 (2022).

30. V. Ballén, C. Ratia, V. Cepas, S. M. Soto, *Enterococcus faecalis* inhibits *Klebsiella pneumoniae* growth in polymicrobial biofilms in a glucose-enriched medium. *Biofouling* **36**, 846–861 (2020).

31. K. Daw, A. S. Baghdayan, S. Awasthi, N. Shankar, Biofilm and planktonic *Enterococcus faecalis* elicit different responses from host phagocytes in vitro. *FEMS Immunol. Med. Microbiol.* **65**, 270–282 (2012).

32. V. Poli, I. Zanoni, Neutrophil intrinsic and extrinsic regulation of NETosis in health and disease. *Trends Microbiol.* **31**, 280–293 (2023).

33. M. Ravindran, M. A. Khan, N. Palaniyar, Neutrophil extracellular trap formation: Physiology, pathology, and pharmacology. *Biomolecules* **9**, 365 (2019).

34. A. W. Martin, H. V. Tartar, The ionization constant of lactic acid, 0–50°, from conductance measurements. *J. Am. Chem. Soc.* **59**, 2672–2675 (1937).

35. M. Jönsson, Z. Saleihan, I. F. Nes, H. Holo, Construction and characterization of three lactate dehydrogenase-negative *Enterococcus faecalis* V583 mutants. *Appl. Environ. Microbiol.* **75**, 4901–4903 (2009).

36. D. Lafrance, L. C. Lands, D. H. Burns, Measurement of lactate in whole human blood with near-infrared transmission spectroscopy. *Talanta* **60**, 635–641 (2003).

37. J.-H. Jeon, C.-W. Hong, E. Y. Kim, J. M. Lee, Current understanding on the metabolism of neutrophils. *Immune Netw.* **20**, e46 (2020).

38. Y. Bao, C. Ledderose, T. Seier, A. F. Graf, B. Brix, E. Chong, W. G. Junger, Mitochondria regulate neutrophil activation by generating ATP for autocrine purinergic signaling. *J. Biol. Chem.* **289**, 26794–26803 (2014).

39. C. Wang, M. J. Taylor, C. D. Stafford, D. S. Dang, S. K. Matarneh, D. E. Gerrard, J. Tan, Analysis of phosphofructokinase-1 activity as affected by pH and ATP concentration. *Sci. Rep.* **14**, 21192 (2024).

40. B. Trivedi, W. H. Danforth, Effect of pH on the kinetics of frog muscle phosphofructokinase. *J. Biol. Chem.* **241**, 4110–4114 (1966).

41. G. P. Dobson, E. Yamamoto, P. W. Hochachka, Phosphofructokinase control in muscle: Nature and reversal of pH-dependent ATP inhibition. *Am. J. Physiol.* **250**, R71–R76 (1986).

42. S. J. Park, C. P. Smith, R. R. Wilbur, C. P. Cain, S. R. Kallu, S. Valasapalli, A. Sahoo, M. R. Guda, A. J. Tsung, K. K. Velpula, An overview of MCT1 and MCT4 in GBM: Small molecule transporters with large implications. *Am. J. Cancer Res.* **8**, 1967–1976 (2018).

43. E. F. Kenny, A. Herzig, R. Krüger, A. Muth, S. Mondal, P. R. Thompson, V. Brinkmann, H. von Bernuth, A. Zychlinsky, Diverse stimuli engage different neutrophil extracellular trap pathways. *eLife* **6**, e24437 (2017).

44. D. W. Edlow, W. H. Sheldon, The pH of inflammatory exudates. *Proc. Soc. Exp. Biol. Med.* **137**, 1328–1332 (1971).

45. H. P. Simmen, J. Blaser, Analysis of pH and pO2 in abscesses, peritoneal fluid, and drainage fluid in the presence or absence of bacterial infection during and after abdominal surgery. *Am. J. Surg.* **166**, 24–27 (1993).

46. T. T. Ward, R. T. Steigbigel, Acidosis of synovial fluid correlates with synovial fluid leukocytosis. *Am. J. Med.* **64**, 933–936 (1978).

47. D. Martínez, M. Vermeulen, A. Trevani, A. Ceballos, J. Sabatté, R. Gamberale, M. E. Álvarez, G. Salamone, T. Tanos, O. A. Coso, J. Geffner, Extracellular acidosis induces neutrophil activation by a mechanism dependent on activation of phosphatidylinositol 3-kinase/Akt and ERK pathways. *J. Immunol.* **176**, 1163–1171 (2006).

48. N. Murata, C. Mogi, M. Tobo, T. Nakakura, K. Sato, H. Tomura, F. Okajima, Inhibition of superoxide anion production by extracellular acidification in neutrophils. *Cell. Immunol.* **259**, 21–26 (2009).

49. O. D. Rotstein, V. D. Fiegel, R. L. Simmons, D. R. Knighton, The deleterious effect of reduced pH and hypoxia on neutrophil migration in vitro. *J. Surg. Res.* **45**, 298–303 (1988).

50. S. Cao, P. Liu, H. Zhu, H. Gong, J. Yao, Y. Sun, G. Geng, T. Wang, S. Feng, M. Han, J. Zhou, Y. Xu, Extracellular acidification acts as a key modulator of neutrophil apoptosis and functions. *PLOS ONE* **10**, e0137221 (2015).

51. M. Behnen, S. Möller, A. Brozek, M. Klinger, T. Laskay, Extracellular acidification inhibits the ROS-dependent formation of neutrophil extracellular traps. *Front. Immunol.* **8**, 184 (2017).

52. D. Stojkov, L. Gigon, S. Peng, R. Lukowski, P. Ruth, A. Karaulov, A. Rizvanov, N. A. Barlev, S. Yousefi, H. U. Simon, Physiological and pathophysiological roles of metabolic pathways for NET formation and other neutrophil functions. *Front. Immunol.* **13**, 826515 (2022).

53. P. Amini, D. Stojkov, A. Felser, C. B. Jackson, C. Courage, A. Schaller, L. Gelman, M. E. Soriano, J.-M. Nuoffer, L. Scorrano, C. Benarafa, S. Yousefi, H.-U. Simon, Neutrophil extracellular trap formation requires OPA1-dependent glycolytic ATP production. *Nat. Commun.* **9**, 2958 (2018).

54. S. Yousefi, D. Stojkov, N. Germic, D. Simon, X. Wang, C. Benarafa, H. U. Simon, Untangling "NETosis" from NETs. *Eur. J. Immunol.* **49**, 221–227 (2019).

55. E. P. Azevedo, N. C. Rochael, A. B. Guimarães-Costa, T. S. De Souza-Vieira, J. Ganilho, E. M. Saraiva, F. L. Palhano, D. Foguel, A metabolic shift toward pentose phosphate pathway is necessary for amyloid fibril- and phorbol 12-myristate 13-acetate-induced neutrophil extracellular trap (NET) formation. *J. Biol. Chem.* **290**, 22174–22183 (2015).

56. H. L. Caslin, D. Abebayehu, A. Abdul Qayum, T. T. Haque, M. T. Taruselli, P. A. Paez, N. Pondicherry, B. O. Barnstein, L. A. Hoeferlin, C. E. Chalfant, J. J. Ryan, Lactic acid inhibits lipopolysaccharide-induced mast cell function by limiting glycolysis and ATP availability. *J. Immunol.* **203**, 453–464 (2019).

57. K. Dietl, K. Renner, K. Dettmer, B. Timischl, K. Eberhart, C. Dorn, C. Hellerbrand, M. Kastenberger, L. A. Kunz-Schughart, P. J. Oefner, R. Andreesen, E. Gottfried, M. P. Kreutz, Lactic acid and acidification inhibit TNF secretion and glycolysis of human monocytes. *J. Immunol.* **184**, 1200–1209 (2010).

58. E. G. Vozza, C. M. Daly, S. A. O'Rourke, H. K. Fitzgerald, A. Dunne, R. M. McLoughlin, *Staphylococcus aureus* suppresses the pentose phosphate pathway in human neutrophils via the adenosine receptor A2aR to enhance intracellular survival. *mBio* **15**, e0257123 (2024).

59. A. F. Carlin, S. Uchiyama, Y.-C. Chang, A. L. Lewis, V. Nizet, A. Varki, Molecular mimicry of host sialylated glycans allows a bacterial pathogen to engage neutrophil Siglec-9 and dampen the innate immune response. *Blood* **113**, 3333–3336 (2009).

60. D. N. Douda, L. Yip, M. A. Khan, H. Grasemann, N. Palaniyar, Akt is essential to induce NADPH-dependent NETosis and to switch the neutrophil death to apoptosis. *Blood* **123**, 597–600 (2014).

61. R. S. Keshari, A. Verma, M. K. Barthwal, M. Dikshit, Reactive oxygen species-induced activation of ERK and p38 MAPK mediates PMA-induced NETs release from human neutrophils. *J. Cell. Biochem.* **114**, 532–540 (2013).

62. A. Llibre, S. Kucuk, A. Gope, M. Certo, C. Mauro, Lactate: A key regulator of the immune response. *Immunity* **58**, 535–554 (2025).

63. E. Khatib-Massalha, S. Bhattacharya, H. Massalha, A. Biram, K. Golan, O. Kollet, A. Kumari, F. Avemaria, E. Petrovich-Kopitman, S. Gur-Cohen, T. Itkin, I. Brandenburger, A. Spiegel, Z. Shulman, Z. Gerhart-Hines, S. Itzkovitz, M. Gunzer, S. Offermanns, R. Alon, A. Ariel, T. Lapidot, Lactate released by inflammatory bone marrow neutrophils induces their mobilization via endothelial GPR81 signaling. *Nat. Commun.* **11**, 3547 (2020).

64. M. Fei, H. Zhang, F. Meng, G. An, J. Tang, J. Tong, L. Xiong, Q. Liu, C. Li, Enhanced lactate accumulation upregulates PD-L1 expression to delay neutrophil apoptosis in sepsis. *View* **5**, 20230053 (2024).

65. A. Z. Zucoloto, J. Schlechte, A. Ignacio, C. A. Thomson, S. Pyke, I.-L. Yu, M. B. Geuking, K. D. McCoy, B. G. Yipp, M. R. Gillrie, B. McDonald, Vascular traffic control of neutrophil recruitment to the liver by microbiota-endothelium crosstalk. *Cell Rep.* **42**, 112507 (2023).

66. D. Awasthi, S. Nagarkoti, S. Sadaf, T. Chandra, S. Kumar, M. Dikshit, Glycolysis dependent lactate formation in neutrophils: A metabolic link between NOX-dependent and independent NETosis. *Biochim. Biophys. Acta Mol. Basis Dis.* **1865**, 165542 (2019).

67. A. D. Wise, E. G. TenBarge, J. D. C. Mendonça, E. C. Mennen, S. R. McDaniel, C. P. Reber, B. E. Holder, M. L. Bunch, E. Belevska, M. G. Marshall, N. M. Vaccaro, C. R. Blakely, D. H. Wellawa, J. Ferris, J. R. Sheldon, J. D. Bieber, J. G. Johnson, L. R. Burcham, A. J. Monteith,

Mitochondria sense bacterial lactate and drive release of neutrophil extracellular traps. *Cell Host Microbe* **33**, 341–357.e9 (2025).

68. S. L. Wong, M. Demers, K. Martinod, M. Gallant, Y. Wang, A. B. Goldfine, C. R. Kahn, D. D. Wagner, Diabetes primes neutrophils to undergo NETosis, which impairs wound healing. *Nat. Med.* **21**, 815–819 (2015).

69. C. C. Murdoch, E. P. Skaar, Nutritional immunity: The battle for nutrient metals at the host–pathogen interface. *Nat. Rev. Microbiol.* **20**, 657–670 (2022).

70. M. Richter, D. Maier-Begandt, J. Jablonska, C. Silvestre-Roig, Sex differences in neutrophil biology. *J. Leukoc. Biol.* **117**, qiaf161 (2025).

71. Y. Lai, L. Cui, G. H. Babunovic, S. M. Fortune, J. G. Doench, T. K. Lu, High-throughput CRISPR screens to dissect macrophage-*Shigella* interactions. *mBio* **12**, e0215821 (2021).

72. W. Zhong, L. Cui, B. C. Goh, Q. Cai, P. Ho, Y. H. Chionh, M. Yuan, A. El Sahili, L. A. Fothergill-Gilmore, M. D. Walkinshaw, J. Lescar, P. C. Dedon, Allosteric pyruvate kinase-based "logic gate" synergistically senses energy and sugar levels in Mycobacterium tuberculosis. *Nat. Commun.* **8**, 1986 (2017).

73. G. M. Dunny, B. L. Brown, D. B. Clewell, Induced cell aggregation and mating in *Streptococcus faecalis*: Evidence for a bacterial sex pheromone. *Proc. Natl. Acad. Sci. U.S.A.* **75**, 3479–3483 (1978).

74. K. M. Hallinen, K. A. Guardiola-Flores, K. B. Wood, Fluorescent reporter plasmids for single-cell and bulk-level composition assays in *E. faecalis*. *PLOS ONE* **15**, e0232539 (2020).

75. J.-H. Ch'ng, M. Muthu, K. K. L. Chong, J. J. Wong, C. A. Z. Tan, Z. J. S. Koh, D. Lopez, A. Matysik, Z. J. Nair, T. Barkham, Y. Wang, K. A. Kline, Heme cross-feeding can augment *Staphylococcus aureus* and *Enterococcus faecalis* dual-species biofilms. *ISME J.* **16**, 2015–2026 (2022).
